# 25 years of *ERMM*

**DOI:** 10.1017/erm.2023.8

**Published:** 2023-04-18

**Authors:** Nicola Curtin

**Affiliations:** Translational and Clinical Research Institute, Newcastle University, Newcastle upon Tyne, UK

This year marks the 25^th^ Anniversary of *Expert Reviews in Molecular Medicine*, or *ERMM* for short. From the outset it was an online-only journal, initially in association with Centre for Applied Research in Educational Technologies and run by an in-house editorial team. At first articles were by invitation only but from 2010 an online submission system was initiated and Professor Tim Cox was appointed as Editor in Chief. Published in the UK it is an international journal, most of its published articles to date have originated in the USA, the UK and Western Europe (29, 23 and 19.4%, respectively) with contributions from China (6.5%), other Asian countries (6.4%), Canada (4.7%), Australia and New Zealand (3.7%), the Middle East (2.6%), Russia and Eastern Europe (2%) Central and South America (1.7%) and Africa (1%) making up the rest. Its impact factor is currently 7.6 with an upward trend over the last 2 years.

I have been the Editor in Chief since 2020 and have been greatly aided by a group of enthusiastic editors from all corners of the earth. The feasibility of working with this international team has been helped by the transition to remote working due to COVID that enabled the normalisation of online team meetings. Thanks to the editorial team we now have several special collections completed and in development and we hope to increase the geographical diversity of our publications.

To celebrate this quarter century event, we are reissuing the 25 most highly cited papers, based on average citations/year. The origins of these articles reflect the geographical diversity of published articles in general with 11 coming from the USA, six from the UK and four from Europe. They perfectly demonstrate the range of subjects for our expert reviews, covering a variety of fields related to the molecular aspects of disease. As cancer is one of the major causes of illness and death, cancer biology features highly in this collection, with six papers reviewing various aspects, two of which focus on cell cycle checkpoints and the others on aetiology, biomarkers and various therapeutic approaches. Reflecting its importance in disease, four of the papers attracting a high level of citations deal with inflammation, including the role of macrophages, the mechanisms of transendothelial flux and cannabinoid receptors in neuroinflammation. Seven of the papers focus on aspects of cell biology/biochemistry relevant to a variety of human diseases of which two focus on chromatin/gene regulation and two on cell signalling. Other themes are neurology, infectious diseases/parasites and antibiotic resistance and toxicology. There is also an interesting article on traditional Chinese medicine.

We hope these articles will be of interest to our readers, and inspire them to submit their own reviews on interesting aspects of molecular medicine to ERMM.

